# Individualizing Therapy in CIDP: A Mini-Review Comparing the Pharmacokinetics of Ig With SCIg and IVIg

**DOI:** 10.3389/fneur.2021.638816

**Published:** 2021-03-08

**Authors:** Said R. Beydoun, Khema R. Sharma, Bassam A. Bassam, Michael T. Pulley, Jeffrey Z. Shije, Ayman Kafal

**Affiliations:** ^1^Neuromuscular Division, Keck School of Medicine of University of Southern California (USC), Los Angeles, CA, United States; ^2^Neurology Department, Miller School of Medicine, University of Miami, Miami, FL, United States; ^3^Neurology Department, University of South Alabama College of Medicine, Mobile, AL, United States; ^4^Department of Neurology, University of Florida College of Medicine, Jacksonville, FL, United States; ^5^CSL Behring, King of Prussia, PA, United States

**Keywords:** CIDP, IVIg, SCIg, IG therapy, dosing strategies, pharmacokinetics, wear-off effect, Ig concentrations

## Abstract

Immunoglobulin (Ig) therapy is a first-line treatment for CIDP, which can be administered intravenously (IVIg) or subcutaneously (SCIg) and is often required long term. The differences between these modes of administration and how they can affect dosing strategies and treatment optimization need to be understood. In general, the efficacy of IVIg and SCIg appear comparable in CIDP, but SCIg may offer some safety and quality of life advantages to some patients. The differences in pharmacokinetic (PK) profile and infusion regimens account for many of the differences between IVIg and SCIg. IVIg is administered as a large bolus every 3–4 weeks resulting in cyclic fluctuations in Ig concentration that have been linked to systemic adverse events (AEs) (potentially caused by high Ig levels) and end of dose “wear-off” effects (potentially caused by low Ig concentration). SCIg is administered as a smaller weekly, or twice weekly, volume resulting in near steady-state Ig levels that have been linked to continuously maintained function and reduced systemic AEs, but an increase in local reactions at the infusion site. The reduced frequency of systemic AEs observed with SCIg is likely related to the avoidance of high Ig concentrations. Some small studies in immune-mediated neuropathies have focused on serum Ig data to evaluate its potential use as a biomarker to aid clinical decision-making. Analyzing dose data may help understand how establishing and monitoring patients' Ig concentration could aid dose optimization and the transition from IVIg to SCIg therapy.

## Introduction

Immunoglobulin (Ig) therapy is recommended in guidelines for the treatment of various neurologic diseases and is a first-line treatment for chronic inflammatory demyelinating polyneuropathy (CIDP), Guillain-Barré syndrome (GBS), multifocal motor neuropathy (MMN), and rescue therapy for worsening myasthenia gravis (MG) (1, 2). Licensed indications for intravenous Ig (IVIg) and subcutaneous Ig (SCIg) varies between products and regions.

The decision between IVIg and SCIg is based on many factors such as long-term side effects, autonomy, disease severity, comorbidities, venous access and patient preference. IVIg is associated with systemic side effects, including rare but serious adverse events (AEs) (3, 4). Serious AEs include hemolysis, thrombotic events and renal failure which can occur with IVIg or SCIg, although to a lesser frequency with the latter. SCIg requires no venous access and has fewer systemic side effects compared with IVIg (5). Switching to self-administered SCIg for maintenance therapy can be more convenient for some patients (6–8).

Tailoring Ig treatment toward individualized regimens may reduce treatment costs without compromising clinical efficacy (9). Dose optimization is, in part, hindered by a lack of reliable biomarkers to measure disease activity and aid clinical monitoring (10, 11). This mini-review discusses treatment individualization for CIDP with an emphasis on the available PK data during the transition from IVIg to SCIg therapy and ongoing treatment optimization.

## CIDP Background

CIDP is an immune-mediated neuromuscular disease characterized by proximal and distal weakness associated with sensory loss and areflexia (12). Pathophysiology is predominantly demyelinating, but if untreated, can progress to secondary axonal loss resulting in irreversible motor deficit (13). CIDP typically follows a progressive course but may have a relapsing-remitting pattern and rarely can present with acute/subacute onset (14).

European Federation of Neurological Societies/Peripheral Nerve Society (EFNS/PNS) guidelines recommend that IVIg is individualized to achieve the lowest effective maintenance dose with periodic attempts to taper the dose in stable patients to determine need for ongoing therapy (2). An initial loading dose of 2 g/kg over 2–5 days followed by lower doses at ~1 g/kg every 3 weeks have been typically adopted as a starting point for IVIg therapy (15, 16).

Ig mechanisms of action in immune-mediated neuropathies are poorly understood but thought to encompass several pathways including Fc receptor blockade, Fcγ receptor modulation, anti-idiotypic antibody binding to autoantibodies, complement neutralization, and cytokine regulation (17, 18). In the absence of disease-specific biomarkers, monitoring serum Ig concentrations has been explored to aid dose optimization by establishing a patient's Ig trough level and tracking this in relation to clinical outcome (19–22). There is no standard Ig trough level threshold for all patients. The optimal trough level for an individual patient can be assessed after disease stabilization and attempts to lower the dose have been attempted. Initial findings support the concept of Ig levels as a biomarker but reinforce the need for therapy individualization (23–25).

## Comparison of SCIg and IVIg in CIDP

SCIg was approved in 2018 for maintenance therapy in IVIg-stabilized adult patients with CIDP and is already widely used in primary immunodeficiency (26–28). IVIg and SCIg are the same therapy via two different modes of administration resulting in different characteristics. The choice between which to opt for in maintenance therapy should be determined in consultation with the patient. Several studies in SCIg allow some comparison of the general characteristics and advantages of IVIg and SCIg (Table 1). No head-to-head trials comparing IVIg and SCIg have been conducted so caution should be exercised when comparing results between studies (4).

**Table 1:** Summarized comparison of IVIg and SCIg characteristics.

	**IVIg**	**SCIg**
INFUSION PRACTICALITIES^*^		
Induction/Loading dose	2 g/kg bw (20 mL/kg) divided over 2–5 consecutive days	N/A—SCIg not approved for induction therapy
Maintenance dose	1 g/kg bw (10 mL/kg) in 1–2 infusions over consecutive days	0.2–0.4 g/kg bw (1–2 mL/kg) in 1–2 infusions
Infusion duration	3–5 h	1–1.5 h
Infusion frequency	Typically, 3–4 weeks	Typically, weekly
Infusion rate	0.3 mL/kg per hour for initial infusion, increasing up to ≤ 4.8 mL/kg per hour, as tolerated^†^	≤ 20 mL/site per hour for initial infusion, increasing up to ≤ 50 mL/site per hour, as tolerated ( ≤ 8 sites simultaneously, typically 2–4 sites used)
Onset of action	1–2 weeks	4 weeks^‡^
Setting	Home, hospital, or infusion clinic	Home, school, work (or other convenient location)
HCP required	Yes	Typically, no
**TYPICAL SAFETY PROFILE**		
Systemic AEs	Yes	Less frequent
Local AEs	Rarely	Yes
Premedication	Yes	Rarely
Venous access	Yes	No
Ig levels	Troughs and peaks	Stable—approaching steady-state
Wear-off effects	Can occur between doses	Rarely due to more frequent infusion
**PATIENT WHO MAY BE MORE SUITABLE FOR IVIg**		
Patients lacking skill, confidence or drive to learn self-administration, including limitations in some elderly patients		
Patients whose compliance for self-administration is in question		
Patients with poor dexterity and lacking a reliable support network		
Patients preferring a clinic setting and/or treatment administered by an HCP		
Patients preferring more infrequent infusions		
Patients with excessive bruising and subcutaneous bleeding tendency		
**PATIENT WHO MAY BE MORE SUITABLE FOR SCIg**		
Patients with poor venous access or those where a port is being considered		
Patients experiencing intolerable side effects with IVIg infusions		
Patients experiencing treatment-related fluctuations between IVIg infusions		
Patients wanting more autonomy, freedom, or flexibility with their infusion location/schedule		
Patients preferring shorter, more frequent infusions		
Patients with comorbidities putting them at higher risk of severe AEs		

* *Assuming use of a 10% IVIg and 20% SCIg formulation—infusion parameters for different formulations may vary, always refer to the product prescribing information*.

† *Infusion rates are product dependent and range from 2 to 8 mg/kg/min*.

‡ *SCIg should be started ≤ 1 week after the final IVIg dose in order to maintain Ig levels and avoid the return of symptoms during transition. AE, adverse event; bw, bodyweight; HCP, healthcare professional; Ig, immunoglobulin; IVIg, intravenous immunoglobulin; SCIg, subcutaneous immunoglobulin*.

### Efficacy

**Figure 1:**
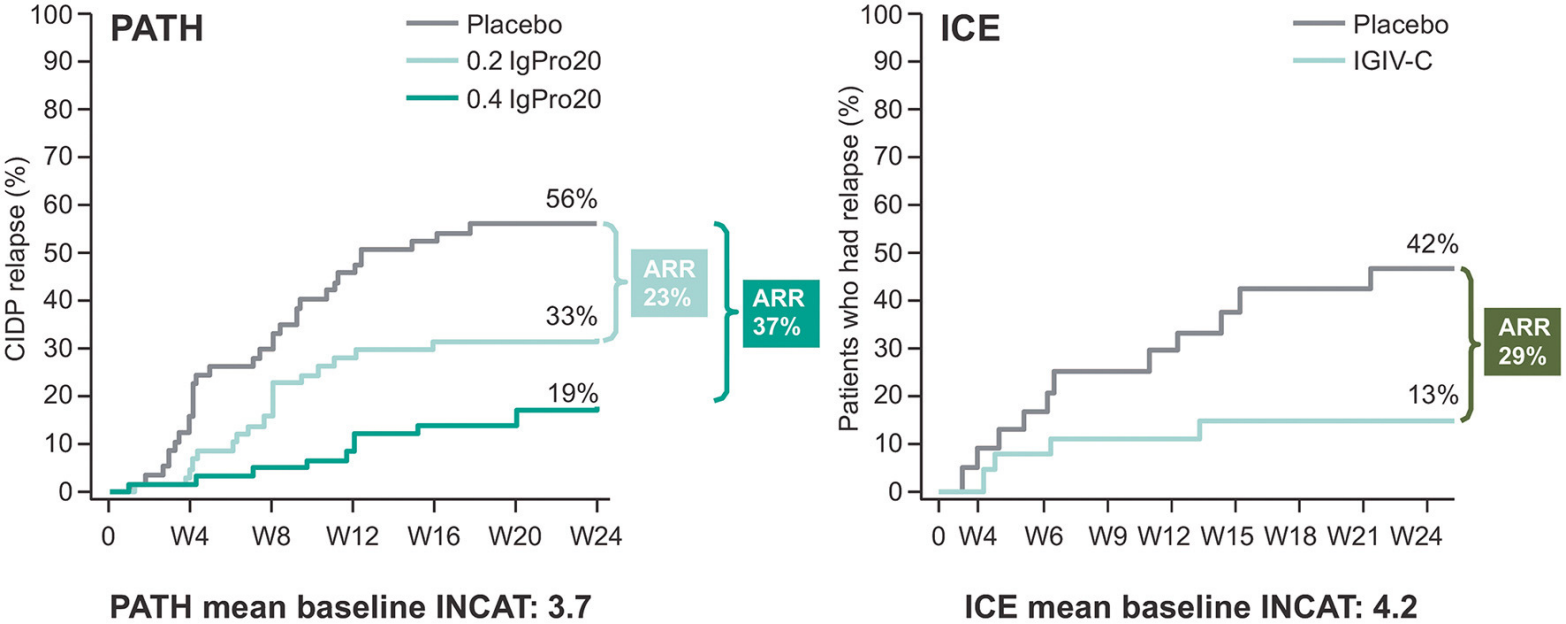
Kaplan-Meier plot showing absolute risk reduction (ARR) in the PATH and ICE trials for CIDP relapse only. CIDP relapse was based on adjusted inflammatory neuropathy cause and treatment (INCAT) score in the randomized SCIg treatment period of the PATH study and the extension phase of the ICE study. Baseline INCAT scores for each trial are also provided. Raw relapse data from ICE and PATH is used for this comparison. Please note PATH and ICE are not head-to head trials. Figure adapted from van Schaik et al. (26) and Hughes et al. (15), copyright Elsevier.

Reports from small SCIg studies in CIDP suggest IVIg and SCIg demonstrate comparable efficacy (4, 7, 29, 30). Additionally, the PATH randomized controlled trial (RCT) (*n* = 172) and its extension (*n* = 82), assessed two doses of SCIg (0.2 g/kg or 0.4 g/kg bodyweight) in IVIg-stabilized patients (26, 31). Significantly fewer patients relapsed on 0.2 g/kg or 0.4 g/kg SCIg vs. placebo in the PATH study, with no statistically significant difference observed between the two doses (26). The number needed to treat (NNT) to prevent relapse was 2.7 with 0.4 g/kg SCIg and 4.2 with 0.2 g/kg SCIg. The NNT for 0.4 g/kg SCIg is similar to an NNT value of 3.03 reported in a systematic review of IVIg studies (5 RCTs, 0.33–0.66 g/kg average weekly dose) in CIDP (32). The clinical relevance of these values cannot be derived due to each RCT using different disability scales and definitions of improvement. The ICE study (*n* = 117) investigated IVIg vs. placebo in CIDP patients (15). Absolute risk reduction (ARR) results from PATH and ICE show similar results (PATH study: 0.2 g/kg SCIg, 23%, and 0.4 g/kg SCIg, 37%; ICE study: IVIg equivalent to 0.3 g/kg weekly dose, 29%) (Figure 1) (15, 26). Comparisons of SCIg and IVIg using data derived from the PATH and ICE studies are made with caution due to their differing designs and study population. The PATH extension confirmed the efficacy of SCIg for an additional 48 weeks (31). Overall, the relapse rate was lowest with 0.4 g/kg SCIg (10%) compared with 0.2 g/kg SCIg (48%).

### Safety

A meta-analysis in 138 patients with CIDP or MMN reported the relative risk of moderate and/or systemic AEs was 28% lower with SCIg compared with IVIg (33). Markvardsen et al. reported the side effects commonly associated with IVIg, such as headache and nausea, were significantly reduced in severity after switching to SCIg in CIDP patients (34). The PATH studies also report a lower frequency of headaches with SCIg compared with a preceding IVIg period (31, 35). In addition, there was no requirement for premedication prior to SCIg infusions (26). No cases of aseptic meningitis, hemolysis, renal failure, or thromboembolic events occurred in patients receiving SCIg—hemolysis was reported in 3.4% of patients during the IVIg period (although none required clinical intervention) (26, 31, 35). Complications with regular venous access is another safety aspect to consider which can be avoided with SCIg (36).

The most common AEs with SCIg are local-site reactions. Reports suggest local reactions are mostly mild/moderate and tend to decrease with subsequent infusions (26, 31). Setting patient expectations prior to SCIg and providing guidance on alleviation methods for local reactions may help patients manage their infusions and ease anxiety (37).

### Patient Benefits

The benefits offered by IVIg and SCIg will vary between patients based on their lifestyle and priorities. SCIg can be self-administered at home providing flexibility and independence. Offering patients more control over their treatment may translate into improved adherence (38). However, some patients may prefer a clinic setting for their infusions or may not be comfortable with self-administration or needles and prefer their infusions to be handled by a healthcare professional. The majority of IVIg maintenance infusions, as per ICE study, were given over 5 h with a mean of 2.7 h (4, 15). SCIg infusions can be conducted in a shorter timeframe while the patient maintains light activity. However, the cumulative time spent on infusions over a 3-weeks period is likely to be near equal for SCIg and IVIg. A patient's ability to learn how to self-administer may be a concern shared by some physicians and patients. However, in the PATH study all patients learned self-administration in four or fewer training sessions with 88% reporting the technique was easy to learn (26). Adequate SCIg training is important to aid a smooth transition to SCIg. Patient factors such as manual dexterity, confidence, compliance, support network, and motivation can affect the success of SCIg and some patients may require additional support/education from their nurse or pharmacy.

### Wear-Off Effects

Reports suggest that the effects of IVIg can “wear off” for some patients before their next dose resulting in a return of symptoms (5). IVIg is typically administered in 3–4 weeks intervals and decreasing Ig concentrations over the following weeks may lead to cyclic treatment-related fluctuations (Supplementary Figure 1). SCIg is administered in smaller weekly doses which better maintains Ig concentration between doses, resulting in narrower peak-to-trough serum levels and may lead to better maintenance of function (39).

## Pharmacokinetic Differences With IVIg and SCIg

Ig has a half-life between 21 and 30 days so typically IVIg infusions are initiated with 3–4-week intervals (39, 40). IVIg delivers Ig directly into the intravascular space, therefore, infusion of a 2 g/kg induction dose can increase Ig concentration within minutes up to 4 times pre-infusion levels, with potential peaks exceeding 30 g/L (41, 42). By competing with the saturable Fc receptors, high concentrations of exogenous Ig from IVIg may redirect endogenous pathologic Ig to lysosomal degradation rather than recycling pathways (43). Ig concentration declines rapidly over the next 48–72 h as it disperses into the extracellular volume (39, 44). The sharp peak in Ig may aid a faster onset of action and functional improvement compared with SCIg (45, 46). This same peak following IVIg infusion can result in systemic AEs and a need for premedication in some patients (45, 47).

SCIg delivers Ig to subcutaneous tissue where diffusion occurs into the bloodstream slowly over 48–72 h (5), resulting in steady-state Ig concentrations that are 12–15% higher than the Ig trough levels typically observed with monthly IVIg infusions (48). Following SCIg infusion, peak Ig concentrations are reached after 36–72 h, with maximum concentrations around ~60% of that typically observed after IVIg infusion (39). This gradual climb toward a lower peak concentration is one reason systemic AEs are less frequent with SCIg (5).

Immunoglobulin bioavailability is expected to be lower for SCIg compared with IVIg (4). Therapeutic Ig degradation in extracellular tissue and binding to extracellular matrix may be the cause of lower bioavailability for SCIg potentially resulting in the need for a higher dose than the equivalent IVIg (4). However, several studies have observed a positive response when adopting an initial 1:1 dose conversion from IVIg to SCIg (7, 49, 50). The PATH study used doses above (0.4 g/kg) and below (0.2 g/kg) the weekly equivalent IVIg dose (0.33 g/kg) and both were efficacious compared with placebo (26).

The importance of maintaining higher trough levels (via SCIg) vs. higher peak levels of Ig (via IVIg) is unclear. One hypothesis is that stable trough levels are important for long-term therapy and better control of systemic AEs, whereas the initial high Ig peak delivered by IVIg may be required to induce improvement rapidly and establish clinical stability (5). However, the exact mechanism and dose response relationship requires better understanding. A study of SCIg in Ig-naïve CIDP patients reported similar improvements in motor function between patients receiving IVIg and SCIg, but the improvement was reached quicker with IVIg (45). SCIg is not approved for induction therapy in CIDP. For patients on maintenance therapy, the PATH extension found that ~9 out of ten patients who relapsed on 0.2 g/kg SCIg could be rescued with 0.4 g/kg SCIg within 4 weeks, which supports using a higher SCIg dose to stabilize patients rather than reverting to IVIg (31). Of the relapses on 0.4 g/kg, 43% recovered within 4 weeks with no dose adjustment. Further data is needed to explore this approach. Many factors should be considered when determining next steps following relapse, such as the severity of the relapse and patient preference. Patients who do not respond to the higher SCIg dose may need to revert to IVIg for maintenance therapy. Maintaining higher steady-state Ig concentrations may avoid the need for rescue boluses of IVIg/SCIg in order to reinstate therapeutic effects (21, 39).

### Tapering Ig Dose

Physicians should consider reducing a patient's dose after a period of clinical stability. Dose adjustments should be based on a combination of neurological examination, patient-reported symptoms and clinical response. Achieving the lowest effective maintenance dose can be via increasing the interval between infusions or reducing the dose (2). However, dose-response studies are limited and report mixed results (4, 19, 21). Recently, the ProCID study, which compared different IVIg dosages during maintenance treatment of CIDP, reported a greater proportion of patients responded at a maintenance dose of 2.0 g/kg compared with 1.0 and 0.5 g/kg (91.7, 79.7, and 64.7%, respectively) (51). During therapy optimization, clinicians should monitor for wear-off effects and be prepared to revert to the prior regimen if necessary (52–55). Lunn et al. proposed infusion interval should be determined based on time to a confirmed relapse and then fixing this interval (54). The patient is then stabilized before attempting 20% dose reductions to determine the minimally effective dose (54). Ig withdrawal attempts can be made on an annual basis in stable patients (54). The authors suggest that lengthening the interval between Ig doses results in unstable Ig concentrations whereas adjusting the dose can achieve more consistent Ig trough levels while quickly determining the minimal dose required (54). One study reported that following therapy optimization 52% of CIDP patients received IVIg at intervals of 10–14 days, and 8% received IVIg at intervals <10 days (21). Smaller intervals between IVIg infusions could help avoid wear-off effects and fluctuating symptoms for some patients. Although, if weekly infusions are required, patients may opt for SCIg as a less invasive and more convenient route of administration. The weekly SCIg dose can be adjusted gradually to assess Ig dependency. SCIg can be useful for minor responsive dosing adjustments to maximize the dose-response relationship (56).

There is a balance between determining Ig dependency, by allowing the patient to deteriorate, and avoiding recurrent relapses which may result in cumulative axonal loss and progressive disability (54, 57). A number of studies are currently investigating different dosing strategies in CIDP. The recently published Gripper study investigated the relationship between Ig levels and clinical response (58). The DRIP study is aiming to determine if more frequent, but lower IVIg doses, leads to more stable Ig concentrations with higher trough levels and clinical efficacy (59).

## PK Studies in CIDP and Other Immune-Mediated Neuropathies

Studies specifically assessing PK parameters in CIDP and IgG therapy are scarce, therefore, a PubMed search was conducted to include PK studies from other immune-mediated neuropathies (Supplementary Table 1). These may offer insights into Ig dosing strategies; however, a limitation is the differences in the underlying mechanisms of different disease states.

### IVIg Studies

Fokkink et al. confirmed the elevated peak serum Ig levels post-IVIg infusion (range 16.7–41.0 g/L) in a cohort of patients with CIDP or MMN within 2 h of receiving IVIg. PK parameters remained constant between infusions in the same patient, but varied considerably between patients (60). Ig concentration after 1 week correlated with grip strength (60). A correlation was also observed in a recent case series between Ig concentration and clinical condition in four CIDP patients on high-dose IVIg treatment, where a decrease in Ig concentration led to symptom fluctuations (24).

A study in eight MMN patients used a smooth transition protocol whereby SCIg was introduced gradually and overlapped with the final IVIg dose (61). Seven patients were successfully switched from IVIg on a 1:1 basis to SCIg (61). The 8th patient had low Ig trough levels and returned to IVIg (61). Researchers suggested low body weight could impact SCIg absorption/distribution (61). In contrast, a review of obesity and Ig concluded that the impact of weight on dosing was not clinically important and should not play a factor in dosing decisions (62). Broyles et al. reported no correlations between Ig variation and weight, clinical response, or total IVIg dose (52).

### SCIg Studies

The PATH study confirmed stable or improved Ig trough levels with weekly SCIg doses over 24 weeks compared with placebo (26). In the PATH extension, changes in Ig trough levels were generally maintained with only a slight decline observed in the 0.2 g/kg SCIg dose (31). Ig trough levels fell noticeably in patients who had relapsed on 0.2 g/kg (−5.3 g/L) compared with patients who relapsed on 0.4 g/kg (−0.2 g/L) (31). Although, 43% of relapses on the 0.4 g/kg dose improved within 4 weeks without intervention (31).

A small RCT in 29 CIDP patients, randomized to either SCIg (1:1 IVIg equivalent dose) or placebo, reported elevated Ig levels in the SCIg group vs. placebo (18.4 vs. 11.3 g/L, respectively (50). These findings were supported by significant differences in muscle strength in the SCIg group compared with placebo (50). However, no relationship was observed between Ig concentration and muscle strength (50).

A retrospective analysis of CIDP patients receiving Ig therapy (IVIg, *n* = 55; SCIg, *n* = 41) found no correlation between Ig concentration and clinical performance (63). The authors concluded Ig level was not a reliable prognostic tool, but group-level data may be confounded by high inter-patient variability in PK and differing infusion intervals (63). Fluctuations in muscle and motor performance measures were unchanged over time in the SCIg group compared with significant fluctuations within the IVIg group (63). The extent of how PK differences contribute to treatment response requires further study, but current findings support SCIg as an advantageous approach to maintaining Ig concentration (44, 64).

### Individualized Dosing

Trials are ongoing on how best to transition from IVIg to SCIg, and updated CIDP treatment guidelines should provide new recommendations. The transition is important as patients' serum Ig concentrations will change from largely variable peaks and troughs to steady-state values, but maintenance of the trough level appears crucial. SCIg may require closer monitoring post-transition to achieve the optimal dose due to inter-patient differences in catabolic pathways, gradual release of Ig from subcutaneous tissue, and Ig clearance mechanisms (42).

There is also the issue of clinical deterioration and relapse, which can be perceived as a risk by patients whose condition has stabilized on IVIg therapy. Use of “smooth transition” protocols may minimize relapse risk and reassure patients who are hesitant to change a treatment regimen that is working (7, 61). Results from a study in MMN show that, despite different PK profiles elicited by IVIg and SCIg, it is possible to avoid clinical deterioration and preserve disability scores by calculating IVIg-equivalent doses for each patient individually and allowing leeway for dose increases (61).

## Conclusions

Ig maintenance therapy can be a continuation of IVIg or a transition to SCIg. What remains unclear is how best to optimize therapy in individual patients. PK assessments show that inter-patient variability is high. A better understanding of the influence of PK parameters on clinical response could aid the process of tailoring Ig therapy. Measuring trough Ig levels can allow determination of optimal Ig dosing for an individual patient whether based on IV or SC administration. In addition, patient factors are an important driver of whether IVIg or SCIg is more suitable for maintenance therapy. Weekly SCIg is a viable alternative for some patients resulting in stable Ig levels while reducing systemic AEs, lowering wear-off risk, and eliminating venous access. Discussions between HCPs and patients to arm them with all the information for either administration route should always take place.

## Author Contributions

SB, KS, BB, MP, JS, and AK equally contributed to the drafting, literature search, and final version of the whole manuscript. All authors reviewed and approved the final manuscript.

## Conflict of Interest

SB had received research grants from Alexion, Argenx, Catalyst, Ra Pharma, and UCB. He has also received honoraria for consulting or speaking for Akcea, Alnylam, CSL Behring, Grifols, Mitsubishi Pharma, and Takeda. BB has received honoraria for consulting or participation in advisory boards for Alexion Pharmaceuticals and CSL Behring. MP has received honoraria for consulting or participation in regional advisory boards from Alexion Pharmaceuticals, Argenx BioProducts Laboratory, Catalyst Pharmaceuticals, CSL Behring, and UCB/Ra Pharma. JS has received honoraria for consulting on an advisory board for Alnylam Pharmaceuticals. AK was a former employee of CSL Behring. The remaining authors declare that the research was conducted in the absence of any commercial or financial relationships that could be construed as a potential conflict of interest.

## Abbreviations

AE: adverse events
ARR: absolute risk reduction
CIDP: chronic inflammatory demyelinating polyneuropathy
cIKS: combined isokinetic strength
EFNS: European Federation of Neurological Societies
FDA: Food and Drug Administration
fSCIG: facilitated SCIg
HCP: healthcare professional
ICE: Immunoglobulin Intravenous CIDP Efficacy
IgG: immunoglobulin G
INCAT: Inflammatory Neuropathy Cause and Treatment
IVIg: intravenous immunoglobulin
NNT: number needed to treat
MG: myasthenia gravis
MMN: multifocal motor neuropathy
MRC: Medical Research Council
OLE: open-label extension
PATH: Polyneuropathy and Treatment with Hizentra
PID: primary immunodeficiency
PK: pharmacokinetics
PNS: Peripheral Nerve Society
Pts: patients
QoL: quality of life
RCT: randomized controlled trial
SC: subcutaneous
SCIg: subcutaneous immunoglobulin
SmPC: Summary of Product Characteristics
US: United States
USPI: United States Prescribing Information.
