# Supplementary figures and images for: Individualizing Therapy in CIDP: A Mini-Review Comparing the Pharmacokinetics of Ig With SCIg and IVIg
Source: Front Neurol. 2021 Mar 8;12:638816. doi: 10.3389/fneur.2021.638816 (PMC7982536; doi:10.3389/fneur.2021.638816)

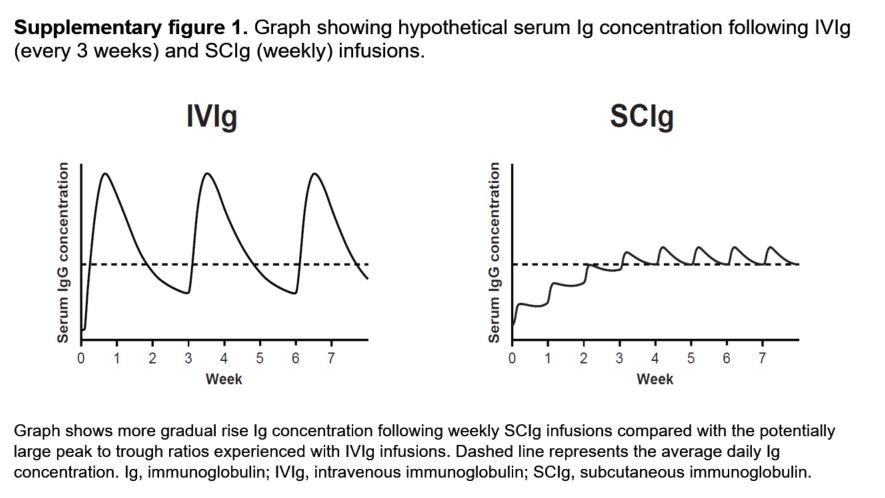

Supplement: Supplementary file 1 [file Image_1.JPEG]
