# Supplementary material for: Individualizing Therapy in CIDP: A Mini-Review Comparing the Pharmacokinetics of Ig With SCIg and IVIg
Source: Front Neurol. 2021 Mar 8;12:638816. doi: 10.3389/fneur.2021.638816 (PMC7982536; doi:10.3389/fneur.2021.638816)
Supplement: Supplementary file 2 [file Table_1.docx]

**Supplementary table 1.** Overview of PK studies with IVIg and SCIg in immune-mediated neuropathies.

| **Study *(indication[s])*** | **Study design** | | **No. patients** | | **Serum Ig data** | **Summary of PK findings** |
| --- | --- | --- | --- | --- | --- | --- |
| **IVIg studies** |  | |  | |  |  |
| **Kuitwaard, et al. 2009**  *(GBS)* | Data collated from two previous RCTs | 174 | | Post-IVIg mean IgG (2 weeks) 18.8 g/L (SD: 5.8)  Mean ΔIgG (2 weeks): 7.8 g/L (range: -5–26)  Post-IVIg mean IgG (4 weeks) 14.0 g/L (SD: 3.1) | | No association between baseline serum IgG levels or at 2 weeks post-IVIg with age, body weight, or symptoms of a recent infection.  Pts with a small ΔIgG (<4 g/L) had more severe disease. These pts may benefit from higher doses or more frequent infusions |
| **Kuitwaard, et al. 2013**  *(CIDP)* | Randomized, controlled double-blind of two IVIg formulations | 25 | | Mean IgG trough 15 g/L (IQR: 13–17)  Mean ΔIgG (5 mins post infusion): 7.8 g/L (IQR: 6–9)  Mean ΔIgG (2 weeks post infusion): 0.1 g/L (range: -0.6–0.7) | | Intra-patient variability was low for IgG levels over serial infusions (indicative of steady state with a constant distribution rate and turnover of IgG without accumulation over time). ΔigG did not correlate with age, sex, bodyweight, lean body mass, muscle strength, or disability |
| **Rajabally, et al. 2013**  *(CIDP)* | Observational study of IVIg-stabilized patients | 15 | | *IgG data not provided* | | Consistent intra-patient IgG levels 14 days post IVIg, but large interpatient variation (no correlation with weight, BMI, BCR or IgG dose) |
| **Hodkinson, et al. 2015**  *(CIDP, MMN, various PIDs)* | Retrospective cohort study in lean and obese patients | 30*  *(8 CIDP & 22 MMN)* | | Mean IVIg dose 1.3 g/kg (range: 0.9–2.1) monthly (range: 14–84)  Mean SCIg dose 0.26 g/kg (range: 0.2–0.4 g/kg weekly (range: 3.5–7)  Mean pre-treatment IgG 10.0 g/L (range: 5.1– 13.4)  Mean IgG trough 18.5 g/L (range: 7.3–33.1) | | Obese pts achieved a higher IgG trough per unit dose of IgG compared with lean pts. It may be possible to reduce the dose in some obese pts without compromising clinical outcome. Dosing should be based on clinical need rather than PK |
| **Debs, et al. 2016**  *(CIDP)* | Case series in homogeneous patients with severe CIDP | | 4 | | Mean IVIg dose 3.5 g/kg (range: 2.7–4.4) monthly  Mean optimum IgG: 32 g/L (range: 29–35)  Mean relapse IgG: 24.5 g/L (range: 19–28) | Pts with severe CIDP responded to high dose IVIg (> 2g/kg) with fractionated doses. Monitoring serum IgG level and its correlation with clinical scores was essential to determine ‘optimum’ and ‘relapse’ IgG concentrations |
| **Fokkink, et al. 2017**  *(CIDP, MMN)* | Observational study of IVIg-stabilized patients | | 15  *(14 CIDP & 1 MMN)* | | Median IVIg dose 30 g (range: 15–70) every 14 days (range: 7–28)  Median IgG peak 25.9 g/L (range: 16.7–41.0)  Median IgG trough 16.1 g/L (range: 9.7– 23.6)  Median IgG half-life 23.1 days (range: 11–60) | PK parameters were constant between two subsequent IVIg infusions in individual pts, but varied greatly between pts.  IgG levels (1 week after infusion) correlated with grip strength |
| **SCIg studies** |  | |  | |  |  |
| **Markvardsen, et al. 2013**  *(CIDP)* | Randomized, double-blind, placebo-controlled | | 29  *(14 on SCIg)* | | Pre-infusion (- 2 weeks) 13.7 ± 2.7 g/L  Post-final IVIg infusion 21.5 ± 4.5 g/L  Study end IgG (Week 12) 18.4 ± 5.2g/L | There was no relationship between plasma levels of IgG and isokinetic strength. However, declines in strength observed between IVIg infusions were eliminated with SCIg. Study suggests 1:1 dose conversion for IVIg to SCIg is effective |
| **Markvardsen, et al. 2017**  *(CIDP)* | Randomized, single blind, cross-over study in treatment-naïve pts | | 19 | | By Week 10  Mean IgG (IVIg) 13.5 ± 2.7 g/L  Mean IgG (SCIg) 12.2 ± 2.8 g/L | A lower baseline level of IgG was associated with a better response to IgG therapy. No correlation between increasing IgG and muscle strength was observed |
| **Christiansen, et al. 2018**  *(CIDP, MMN)* | Prospective open label follow-up trial | | 17  *(12 CIDP, 2 MADSAM, & 3 MMN)* | | Average IgG (IVIg) 21.3 g/L (CI: 19.5–23.2)  Average IgG (SCIg) 19.3 g/L (CI: 17.8–20.7) | Transition to SCIg preserved overall strength, disability and QoL, but reduced fluctuations in physical performances (40-MWT & 9-HPT) |
| **van Schaik, et al. 2018**  *(CIDP)* | Randomized, double-blind, placebo-controlled | | 172  *(115 on SCIg)* | | Last post-dose observational  Mean IgG (low-dose SCIg) 15.4 ± 3.1 g/L  Mean IgG (high-dose SCIg) 20.4 ± 3.2 g/L | Compared with baseline, IgG levels increased in the high-dose group and remained stable in the low-dose group. |
| **Van Schaik, et al. 2019**  *(CIDP)* | Prospective, open-label extension study | | 82 | | End of study (non-relapsers)  Mean IgG (low-dose SCIg) 16.0 ± 2.6 g/L  Mean IgG (high-dose SCIg) 20.3 ± 3.7 g/L | IgG trough levels declined in low-dose pts but were stable in high-dose patients. In pts that relapsed the decrease was greater in low-dose pts. |
| **Markvardsen, et al 2019**  *(CIDP)* | Retrospective correlational analysis [meta-analysis] | | 96  *(55 on IVIg; 41 on SCIg)* | | Combined IgG data from 5 previous studies converted into % difference | No relationship between absolute IgG concentration at given timepoint or ΔIgG and the % muscle strength (IKS). |

*Data from patients with PIDs excluded from this table. 9-HPT, 9-hole peg test; 40-MWT, 40-meter walk test; BCR, best clinical response; BMI, body mass index, CI, confidence interval; CIDP, chronic inflammatory demyelinating polyneuropathy; IgG, immunoglobulin G; IVIg, intravenous immunoglobulin; MADSAM, Multifocal acquired demyelinating sensory and motor polyneuropathy; MMN, multifocal motor neuropathy; MRC, Medical Research Council; PID, primary immunodeficiency; PK, pharmacokinetic; pts, patients, SCIg, subcutaneous immunoglobulin. Studies were identified from a PubMed literature search using the following terms: (immunoglobulin G OR IgG) AND (chronic inflammatory demyelinating polyneuropathy OR CIDP) AND ((pharmacokinetic OR PK) OR (pharmacodynamic OR PD)) and (immunoglobulin G OR IgG) AND ((multi-focal motor neuropathy OR MMN) OR (Guillain-Barré syndrome OR GBS) OR (myasthenia gravis OR MG)) AND ((pharmacokinetic OR PK) OR (pharmacodynamic OR PD)); reviews were excluded. Studies collated to provide overview of published PK data and any comparisons drawn between studies should be made with caution due to differing neurologic disease states, study designs, and population
